# Elastography in the assessment of the Achilles tendon: a systematic review of measurement properties

**DOI:** 10.1186/s13047-023-00623-1

**Published:** 2023-04-27

**Authors:** Tiziana Mifsud, Alfred Gatt, Kirill Micallef-Stafrace, Nachiappan Chockalingam, Nat Padhiar

**Affiliations:** 1 Faculty of Health Sciences, University of Malta, Msida, Malta; 2 Department of Orthopaedics, Trauma and Sports Medicine, Mater Dei Hospital, L-Imsida, Malta; 3 Centre for Biomechanics and Rehabilitation Technologies, Staffordshire University, Stoke On Trent, Staffordshire, UK; 4 Centre for Sports &Exercise Medicine, St Bartholomew’s & The London School of Medicine & Dentistry, William Harvey Research Institute, Queen Mary University of London, London, UK

**Keywords:** Elastography, Reliability, Validity, Responsiveness, Achilles tendinopathy

## Abstract

**Background:**

Managing and rehabilitating Achilles tendinopathy can be difficult, and the results are often unsatisfactory. Currently, clinicians use ultrasonography to diagnose the condition and predict symptom development. However, relying on subjective qualitative findings using ultrasound images alone, which are heavily influenced by the operator, may make it difficult to identify changes within the tendon. New technologies, such as elastography, offer opportunities to quantitatively investigate the mechanical and material properties of the tendon. This review aims to evaluate and synthesise the current literature on the measurement properties of elastography, which can be used to assess tendon pathologies.

**Methods:**

A systematic review was conducted according to Preferred Reporting Items for Systematic Reviews and Meta-Analyses guidelines. CINAHL, PubMed, Cochrane, Scopus, MEDLINE Complete, and Academic Search Ultimate were searched. Studies assessing the measurement properties concerning reliability, measurement error, validity, and responsiveness of the instruments identified in healthy and patients with Achilles tendinopathy were included. Two independent reviewers assessed the methodological quality using the Consensus-based Standards for the Selection of Health Measurement Instruments methodology.

**Results:**

Out of the 1644 articles identified, 21 were included for the qualitative analysis investigating four different modalities of elastography: axial strain elastography, shear wave elastography, continuous shear wave elastography, and 3D elastography. Axial strain elastography obtained a moderate level of evidence for both validity and reliability. Although shear wave velocity was graded as moderate to high for validity, reliability obtained a very low to moderate grading. Continuous shear wave elastography was graded as having a low level of evidence for reliability and very low for validity. Insufficient data is available to grade three-dimensional shear wave elastography. Evidence on measurement error was indeterminate so evidence could not be graded.

**Conclusions:**

A limited number of studies explored quantitative elastography on Achilles tendinopathy as most evidence was conducted on a healthy population. Based on the identified evidence on the measurement properties of elastography, none of the different types showed superiority for its use in clinical practice. Further high-quality studies with longitudinal design are needed to investigate responsiveness.

**Supplementary Information:**

The online version contains supplementary material available at 10.1186/s13047-023-00623-1.

## Background

The Achilles tendon is the most commonly injured tendon in the body [1]. Achilles tendinopathy is common and in particular, mid-portion (free tendon) Achilles tendinopathy which can affect any adult, whether sedentary or involved in sport or physical activity [1, 2]. There is a higher prevalence in high-impact tendon loading sports, such as long-distance running and football [2]. The aetiology and mechanism of this disorder are largely inconclusive and disputed. However, it is largely agreed that it is not an inflammatory condition but more degenerative with failure to repair [3, 4]. The presence of neovascularity seems to be the source of the pain and in the last two decades, it is also the focus of targeted treatment [5–7].

Achilles tendinopathy can be difficult to manage and rehabilitate, taking a prolonged period to obtain positive results in pain reduction and normal functioning [8, 9]. Although various treatment modalities are available with limited evidence on the mode of action, there are no established monitoring instruments and associated clinical protocols. Whilst ultrasonography is used clinically to diagnose and predict the development of symptoms [10], its role in detecting change in follow-up improvement during rehabilitation remains debatable. During the last consensus on the reported outcome measures in tendinopathy clinical trials (ICON 2019) [11], the panel of experts failed to reach an agreement on the sonographic structural changes as an important consideration in tendinopathy. Moreover, the sensitivity of standard sonography is limited, because conventional sonographic signs are missing in a relevant number of symptomatic individuals [12] and full symptomatic recovery does not ensure full recovery of muscle–tendon structure and function [13, 14].

With improvements in ultrasound technology, elastography has emerged as a potential measurement instrument offering opportunities to quantitatively investigate the mechanical and material properties [15] within the tendon. Elastography research has shown rapid growth in the past 10 years and has been used to understand how loading [16] ageing [17] and different treatments [18] are affecting tendon recovery [19, 20]. Elastography has been claimed as having better sensitivity and specificity than ultrasonography in the diagnosis and monitoring of tendinopathies. Different types and modes of action of elastography exist, but their effectiveness in assessing Achilles tendon patients with tendinopathy has not been evaluated in a systematic review. This manuscript does not aim to provide a detailed explanation of all the different modes of action for each elastography technique, but the reader is referred to other narrative reviews [21, 22] for further understanding.

Knowledge of measurement properties helps to inform the clinician and researchers in the choice of the most appropriate equipment to be used whilst achieving more accurate, reliable, and valid results. Using measurement instruments with poor measurement properties increases the risk of bias in results obtained and may fail to detect a true change when assessing different treatment modalities and monitoring rehabilitation [23]. Considering the continuous encouragement of clinicians to use evidence-based practice and perform measurements that can monitor recovery, a better understanding of the technologies and techniques of elastography is needed. A systematic evaluation of the current knowledge of quantitative elastography used on healthy and Achilles tendinopathy during both static and dynamic functioning will help to provide evidence for its use in clinical practice. The aim of the reported work is to identify, evaluate and synthesize the current literature on elastography used on healthy and tendinopathic Achilles tendons in order to provide evidence for its use in clinical practice. Measurement properties of reliability, measurement error, validity, and responsiveness will be considered.

## Method

This systematic review was designed and conducted according to guidelines outlined by Preferred Reporting Items for Systematic Reviews and Meta-Analyses (PRISMA) [24].

### Information source and search strategy

Electronic databases including EBSCO, CINAHL, PubMed, Cochrane, Scopus, MEDLINE Complete and Academic Search Ultimate were searched by one investigator (TM). A broad search strategy was developed using both free-text terms and MeSH index terms using a combination of keywords as seen in Appendix 1. To identify studies of measurement properties a validated methodological search filter was used (https://www.cosmin.nl/tools/PubMed-search-filters/). The full search strategy is available in Appendix 2. In addition, reference lists of the included and possible eligible articles were also hand searched and scrutinized to identify any additional studies. No restriction was made on the publication year, however, only articles published in the English language were included. Retrieved references were exported to a reference manager to identify and remove any duplicates present.

### Eligibility criteria and study selection

Two independent reviewers (TM and AG) screened the title and abstract of available articles to identify studies that used elastography to measure the mechanical properties of the Achilles tendon, according to the following inclusion and exclusion criteria as listed in Table 1. The full text of the shortlisted papers was then reviewed to obtain a final set of articles. Any disagreement over the eligibility of studies was resolved through discussion amongst both reviewers. If an agreement was not reached, a third reviewer was consulted (NP). When abstracts and full texts of potential inclusion articles were not found, the authors of these articles were contacted.

**Table 1:** List of inclusion and exclusion criteria

	**Inclusion Criteria**	**Exclusion Criteria**
**Population**	Participants over the age of 18	Participants with insertion AT, history of tendon rupture, past tendon surgery or other causes of heel pain
	Physically active and sedentary	
	Healthy participants and those identified as having mid-portion Achilles tendinopathy	
**Study type**	Studies of any design, especially tool development or validation studies	Studies that only used elastography as an outcome measurement without taking into consideration the measurement properties
	The measurement properties of different elastography methods included reliability, measurement error, validity and responsiveness	Non-peer-reviewed papers, such as editorials or letters to the editor
	Studies investigating the Achilles tendon together with other tendons or muscles but providing separate results for different areas	Studies investigating the Achilles tendon together with other tendons or muscles but presenting results for the whole cohort
	Scientific papers in peer-reviewed journals	In vitro or cadaveric studies

### Data extraction

Data were extracted by the primary reviewer (TM) for each of the included studies. The secondary reviewer checked the extracted data. Data extracted included: the study design, setting, method of assessment, population characteristics, outcome measures, equipment used and specifications, statistical results, measurement properties focusing on reliability, measurement error, validity, responsiveness, and the limitations of the study. When reliability or measurement errors were investigated, the seven elements that construct the research question were also extracted as per guidelines by the COSMIN Manual 2021 (Appendix 3).

### Methodological quality evaluation of the studies

The included articles were assessed for methodological quality by the two independent reviewers, using the COSMIN methodology [25, 26] consisting of three sub-steps. First, the methodological quality of every single study was assessed using the COSMIN Risk of Bias (ROB) checklist to assess reliability, measurement error, validity, and responsiveness for each study, respectively. Each standard was rated on a 4-point scale as very good, adequate, doubtful, or inadequate quality. To determine the overall quality rating of every article, the lowest rating of any standard was taken [26].

Secondly, the statistical results of every study were rated against the criteria for good measurement properties as sufficient ( +), insufficient (-), or indeterminate (?) [26]. Reliability was rated as sufficient if the results of ICC were ≥ 0.70 [26], while for measurement error, the smallest detectable change or the limits of agreement were smaller than the minimum important change. Criterion and construct validity were rated as sufficient if the results were in accordance with the predefined hypothesis by the review team. The correlation between compared instruments (convergent validity) had to be ≥ 0.70 or show no significant differences between instruments. The comparison between groups that were expected to be different (discriminative validity) had to be significantly different. Responsiveness was rated as sufficient when results obtained were able to detect a significant important change over time [26].

Finally, the quality of the evidence was graded (high, moderate, low, or very low evidence) using the modified Grading of Recommendations Assessment, Development, and Evaluation (GRADE) approach. This approach takes into consideration the methodological quality of the studies (COSMIN score), inconsistency of the results per measurement property between the different articles, imprecision, including the total sample size of the available studies and indirectness involving evidence from different populations than the population of interest in this review. Multiple studies were only combined when the same measurement property was evaluated for specific types of elastography. Moreover, when results across reported test conditions were consistent, these results were summarized to determine the overall evidence of the measurement properties. Measurement properties from studies that were rated ‘doubtful or inadequate’ on the COSMIN ROB were not eligible to be combined in evidence synthesis. Any discrepancies between reviewers were discussed and resolved via consensus with a third reviewer (NP).

## Results

### Search strategy

**Fig. 1:**
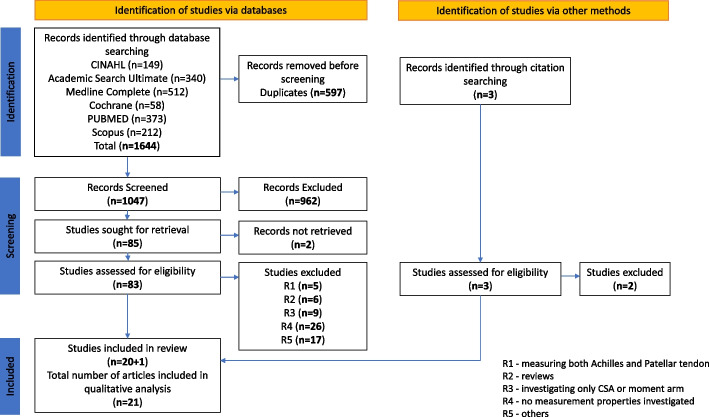
PRISMA flow chart

The literature search was conducted on 10th September 2020 and updated on 15th January 2022. It yielded 1644 articles, of which 597 were duplicates and therefore were removed. Of the 1047 article titles and abstracts which were screened, 83 articles were eligible for full-text assessment. Of these 83 articles, 20 articles were found to be appropriate for inclusion, together with another article identified through citation searching. Thus, analysis was conducted on a total of 21 articles. The full PRISMA flow diagram summarizing the screening process and results are provided in Fig. 1.

### Study characteristics

A summary of study characteristics, including participants’ demographics, are provided in Table 2. The study populations included mainly healthy populations except in five articles [27–31] that investigated patients with midportion Achilles tendinopathy. Healthy asymptomatic participants were younger than participants with Achilles tendinopathy in almost all articles. The sample size of the included studies ranged from three [32] to 326 [27], either investigating one limb or bilateral. Detailed justifications for sample sizes were not provided in all the studies. Half of the articles did not report any information related to the participant’s physical activity levels and the other half had a range of levels from normal daily walking to participation in recreational sports. None of the articles included professional athletes. A cross-sectional research design was implemented in most studies, with only a few prospective longitudinal studies included. In Table 3, the different instrument specifications and probe use are reported as different elastography machines should be considered when collating data for the best evidence available.

**Table 2:** Demographic Data

Measurement instrument	Authors & year	Participants Characteristics			
		**Gender (Male: Female)**	**Age range Mean ± SD (years)**	**Study population**	**Physical activity level**
**Strain elastography**	Drakonaki et al., 2009 [33]	13 M:12F	range 20–52 38.8 ± 5.0	Healthy tendon	NR
	Ooi et al., 2015 [27]	90 M:30F	44.9 ± 13.6	Healthy tendon	occasional recreational sports
			45.2 ± 13.1	Achilles tendinopathy	occasional recreational sports
	Yamamoto et al., 2016 [34]	16 M:9F	range 21–38 28.0	Healthy tendon	< 1 day per week
	Schneebeli et al., 2016 [35]	10 M:14F	28.8 ± 8.8	Healthy tendon	NR
	Payne et al., 2017 [32]	4 M:4F	25.5 ± 2.5	Healthy tendon	normal daily walking
	Schneebeli et al., 2019 [36]	18 M:19 F	27.1 ± 7.0	Healthy tendon	not controlled
	Schneebeli et al., 2021 [37]	12 M:8F	28.9 ± 4.16	Healthy tendon	NR
**Shear wave elastography—velocity**	Aubry et al., 2013 [38]	37 M:43F	range 20–83 45.4	Healthy tendon	37 not active, 43 > 1 h per week
	DeWall et al., 2014 [39]	5 M:5F	26.7 ± 4.1	Healthy tendon	NR
	Aubry et al. 2015 [28]	68 M:12F	range 31–57	Healthy tendon	NR
		19 M:6F	range 46–63	Achilles tendinopathy	NR
	Dirrichs et al., 2016 [29]	26 M:15F	42 ± 13.4	Healthy tendon & Achilles tendinopathy	NR
	Fu et al., 2016 [40]	165 M:161F	range 19–88 48.8 ± 17.1	Healthy tendon	NR
	Payne et al., 2017 [32]	7 M:7F	26.5 ± 3.8	Healthy tendon	normal daily walking
	Coombes et al., 2018 [30]	11 M:17F	38.3 ± 16.7	Healthy tendon	physically active
		13 M:9F	47.5 ± 11.4	Achilles tendinopathy	
**Shear wave elastography—modulus**	Helfenstein-Didier et al., 2016 [41]	12 M:0F	23.2 ± 3.3	Healthy tendon	NR
	Lima et al., 2017 [42]	24 M:0F	28.0 ± 2.0	Healthy tendon	various levels but consistent during testing
	Zhou et al., 2019 [43]	14 M:6F	22.5 ± 3.0	Healthy tendon	normal daily walking
	Gatz et al., 2021 [31]	18 M:19F	range 21–69 37.0 ± 14.0	Healthy tendon	3.2 ± 2.4 h active per week
		16 M:22F	range 22–75 46.0 ± 14.0	Achilles tendinopathy	2.3 ± 2.7 h active per week
**cSWE**	Suydam et al., 2015 [44]	29—gender NR	29.0 ± 9.5	Healthy tendon	NR
	Corrigan et al., 2019 [45]	11 M:9F	29.0 ± 4	Healthy tendon	NR
**3D SWE**	Götschi et al., 2021 [46]	6 M:4F	28.1 ± 3.0	Healthy tendon	physically active > 30 min of moderate activity per week

*M* Male, *F* Female, *NR* Not reported

**Table 3:** Elastography equipment specifications used

Measurement instrument	Authors & year	Machine	Probe	Reference material
**Strain elastography**	Drakonaki et al., 2009 [33]	HV900, Hitachi Medical Corporation	6-14MHz linear transducer	Kager’s fat pad
	Ooi et al., 2015 [27]	Philips iU22 Philips Healthcare, Bothell	5-17MHz linear probe	Kager’s fat pad
	Yamamoto et al., 2016 [34]	HV900; Hitachi Aloka Medical Corporation	6–14MHz linear probe	acoustic coupler—elastomer resin
	Schneebeli et al., 2016 [35]	MyLab ClassC, Esaote, Genoa, Italy	3–13 MHz linear probe	external reference material
	Payne et al., 2017 [32]	Siemens ACUSON S2000™ HELX EVOLUTION	linear 5-14MHz probe	no reference material was used. For analysis, raw data measuring the actual displacement used
	Schneebeli et al., 2019 [36]	MyLab Class C, Esaote, Genova	linear 3-13MHz probe	external reference material
	Schneebeli et al., 2021 [37]	1. Resona 7, Mindray, Shenzhen; 2.Aplio 500, Toshiba Medical Systems Corp.; 3.Aixplorer, SuperSonic Imagine, Aix-En-Provence,	linear—1.6-14MHz; 2. 5-14MHz; 3. 4–15MHz probe	acoustic coupler—elastomer resin
**Shear wave elastography—velocity**	Aubry et al., 2013 [38]	SuperSonic Imagine, Aix-En-Provence, France)	12-MHz superficial Linear transducer	NA
	DeWall et al., 2014 [39]	SupersonicImagine;Aix-en-Provence,France;software version 5	linear array transducer(L15-4)	NA
	Aubry et al. 2015 [28]	Aixplorer, SuperSonic Imagine, Aix-en-Provence, France	12-MHz superficial linear transducer	NA
	Dirrichs et al., 2016 [29]	Aixplorer, SuperSonic Imagine, Aix-en-Provence, France	linear 15 MHz transducer (SuperLinear SL15-4, SuperSonic Imagine	NA
	Fu et al., 2016 [40]	Acuson S3000 ultrasound system (Siemens Medical Solutions VTIQ; Siemens Medical Solutions, Malvern, PA)	9L4 linear transducer	NA
	Payne et al., 2017 [32]	Siemens ACUSON S2000™ HELX EVOLUTION	linear 4-9MHz probe	NA
	Coombes et al., 2018 [30]	Aixplorer version 8.2; Supersonic Imagine, Aix-en-Provence, France)	50 mm linear transducer (15–4 MHz)	NA
**Shear wave elastography—modulus**	Helfenstein-Didier et al., 2016 [41]	AIXPLORER v8, Supersonic Imagine, Aix-en-Provence	superlinear 14–5/38 mm	NA
	Lima et al., 2017 [42]	AIXPLORER v.9, Supersonic Image, Aix-en-Provence	superlinear 4-15 MHz and 2-10 MHz probes	NA
	Zhou et al., 2019 [43]	AIXPLORER, Supersonic Imagine, Aix-en-Provence	10-2MHz/40 mm linear array transducer	NA
	Gatz et al., 2021 [31]	Aixplorer, Super-Linear SL 18–5; Supersonic Imagine	superlinear 18–5	NA
**cSWE**	Suydam et al., 2015 [44]	MDP, Ultrasonix, Vancouver	linear 38 mm and external actuator	NA
	Corrigan et al., 2019 [45]	SonixMDP Q + , Ultrasonix, Vancouver	linear L14-5/38mm and external actuator	NA
**3D SWE**	Götschi et al., 2021 [46]	Aixplorer Ultimate SuperSonic Imagine	super linear 18-5MHz/50mm	NA

### Quality of review articles—methodological quality

Results were grouped according to the type and mode of action of elastography for better homogeneity and consistency within the results. Of the 21 articles included, seven articles investigated strain elastography where strain ratio was calculated as a semi-quantitative measure of stiffness [27, 32–37], eleven investigated shear wave imaging presenting results as either shear wave velocity (SWV) [28–30, 38–40, 47] or modulus [31, 41–43], two investigated continuous shear wave elastography (cSWE) [44, 45], while the last article assessed three-dimensional shear wave elastography (3D SWE) [46]. A summary of the overall quality ratings for the measurement properties of each elastography method and their statistical results are reported in Table 4 and Table 5. Table 4 presents extracted data for the different types of reliability and measurement error (intra-rater, inter-rate and inter-session), while Table 5 presents the validity and responsiveness.

**Table 4:** Reliability and Measurement error results

Outcome Measurement Instrument	Author and year	Reliability					Measurement Error		
		**Study population**	**Design**	**Cosmin Rate**	**Statistical Results**	**Measurement property ratings based on statistical results**	**Cosmin Rate**	**Statistical Results**	**Measurement property ratings based on statistical results**
**Strain elastography**	Drakonaki et al., 2009 [33]	25 bilateral	Inter-rater same day	Adequate	ICC T = 0.41 L = 0.51	-	Adequate	CV T = 30% L = 29.6%	?
			Intra rater same day		ICC T = 0.41,0.45 L = 0.78,0.66	+		CV T = 39% L = 30.50%	?
	Ooi et al., 2015 [27]	10 bilateral	Inter rater different days	Very good	ICC = 0.79	+	NA	NA	NA
			Intra rater different days	Very good	ICC = 0.87	+			
	Yamamoto et al., 2016 [34]	25 bilateral	Inter rater same day	Doubtful	Spearman = 0.61	NA	Adequate	SEM = 0.06,0.07	?
			Intra rater same day	Adequate	ICC = 0.93 ICC = 0.87	+			
	Schneebeli et al., 2016 [35]	24 bilateral	Intra rater same day	Adequate	ICC relaxed = 0.87 ICC contracted = 0.94	+	NA	NA	NA
	Payne et al., 2017 [32]	8 right	Intra rater same day Intra rater different days	Adequate	ICC T = 0.00–0.11, L = 0.01–0.11 both foot position	-	Adequate	CV L = 80.8%-111.5% T = 53.6—112.4%	?
**Shear wave elastography—velocity**	Aubry et al., 2013 [38]	30 bilateral	Inter-rater same day	Doubtful	ICC L = -0.011–0.46, T-0.062–0.29	NA	NA	NA	NA
	DeWall et al., 2014 [39]	10 feet side not reported	Inter-rater	Doubtful	CV = 0.156	NA	NA	NA	NA
	Fu et al., 2016 [40]	326 bilateral	Inter-rater same day	Adequate	ICC L = 0.923, T = 0.870	+	NA	NA	NA
	Payne et al., 2018 [47, 48]	3 dominant foot	Inter-rater same day	Very good	ICC T-0.70 L-0.80	+	Very good	SEM T = 0.19–0.26 L = 0.19–0.27 CV T = 3.9–6.3% L = 2.9–5.2%	?
		14 dominant foot	Intra rater same day Intra rater different days		ICC T = 0.62–0.85, L = 0.45–0.71	-			
	Coombes et al., 2018 [30]	6 bilateral	Inter session different days	Very good	ICC = 0.71	+	NA	NA	NA
	Götschi et al., 2021 [46]	10 right	Inter rater different days	Adequate	ICC = 0.455	-	Adequate	SEM = 1.043 m/s	?
			Inter session different days	Adequate	ICC = 0.591	-	Adequate	SEM = 1.068 m/s	?
**Shear wave elastography—modulus**	Helfenstein-Didier et al., 2016 [41]	7 right	Intra session same day	Adequate	ICC s = 1.0 kPa c = 0.42–0.85 MPa	-	Adequate	SEM s = 1.37–2.14 kPa, c = 3.5–10.69 MPa	?
	Lima et al., 2017 [42]	24 bilateral	Intra session different days	Doubtful	ICC = 0.82–0.93	NA	Doubtful	CV = 23–25%	NA
			Inter sessions different days		ICC = 0.42–0.60 to isometric contraction				
	Zhou et al., 2019 [43]	20 dominant foot	Inter rater different days	Very good	ICC = 0.76–0.94	+	Very good	SEM = 14.38–15.78 kPa	+
			Intra rater different days		ICC = 0.77–0.93	+		SEM = 11.87–21.75 kPa	+
**cSWE**	Suydam et al., 2015 [44]	29 bilateral	Intra rater same day	Adequate	ICC s = 0.875, v = 0.876	+	Adequate	SEM s = 3.8 kPa v = 6.8Pas	+
	Corrigan et al., 2019 [45]	20 either right or left	Intra rater same day Intra rater different days	Adequate	ICC s = 0.697, v = 0.856, d = 0.855	+	Adequate	SEM s = 8.28 kPa, v = 4.79 Pas, d = 46.72 kPa	?
**3D SWE**	Götschi et al., 2021 [46]	10 right	Inter rater different days	Adequate	ICC = 0.436	-	Adequate	SEM = 0.553 m/s	?
			Inter sessions different days		ICC = 0.591	-		SEM = 0.505	?

*NA* Not applicable, *ICC* Intraclass correlation coefficient, *CV* Coefficient of variance, *SD* Standard deviation, *SEM* Standard error of measure, *L* Longitudinal, *T* Transverse, *c* Compression modulus, *s* Shear modulus, *v* Viscosity modulus, *d* Dynamic modulus

**Table 5:** Validity and responsiveness results

**Outcome Measurement Instrument**	**Author and year**	**Construct validity**				**Responsiveness**	
		**Study population**	**Cosmin Rate**	**Statistical Results**	**Measurement property ratings based on statistical results**	**Cosmin Rate**	**Measurement property ratings based on statistical results**
**Strain elastography**	Ooi et al., 2015 [27]	120 control120 with AT	Very good	ultrasonography vs strain ratio spearman = 0.81 strain ratio vs VISA-A *r* = -0.62, *p* < 0.001	+	NA	NA
	Yamamoto et al., 2016 [34]	50 bilateral	Very good	No significant difference between age groups except for the 30age group	-	Very good	+
	Schneebeli et al., 2019 [36]	37 bilateral	Very good	Friedman test *p* < 0.01 significant difference	+	NA	NA
	Schneebeli et al., 2021 [37]	20 bilateral	Very good	Friedman test *p* < 0.01 significant difference	+	NA	NA
**Shear wave elastography—velocity**	Aubry et al., 2013 [38]	80 bilateral	Adequate	Univariate analysis	+	NA	NA
	DeWall et al., 2014 [39]	10	Adequate	Three-way ANOVA for posture, path & region	+	NA	NA
	Aubry et al., 2015 [28]	30 pathological, 180 healthy	Adequate	Normal had significantly lower mean velocity than AT axial SW for plantarflexion (*P* < .001), at sagittal SW for 0° (*P* = .001), and at axial SW for 0° (*P* = .0026)	+	NA	NA
	Fu et al., 2016 [40]	326 bilateral	Adequate	Pearson—no significant correlation of SW velocity with age	-	NA	NA
	Dirrichs et al., 2016 [29]	41 bilateral	Adequate	SW velocity correlates to VISA-A	+	NA	NA
	Coombes et al., 2018 [30]	50 right or left, healthy and AT	Adequate	AT lower SW velocity at insertion (*P* < .001), but not mid-tendon region (*P* = .456)	+	NA	NA
**Shear wave elastography—modulus**	Helfenstein-Didier et al., 2016 [41]	10 right	Adequate	Pearson = 0.844 SW elastography to dispersion analysis confirming the guided wave propagation	+	NA	NA
	Lima et al., 2017 [42]	24 healthy- bilateral	Adequate	Pearson = 0.00–0.041 no correlation to isometric contraction	-	NA	NA
	Schneebeli et al., 2021 [37]	20 bilateral	Very good	Friedman test *p* < 0.05	-	NA	NA
	Gatz et al., 2021 [31]	75	Adequate	Diagnostic—No significant difference (*P* = .062-.994) in favour one modality	+	Very good	
**cSWE**	Suydam et al., 2015 [44]	29 bilateral	Adequate	Pearson < 0.12 between shear modulus and MVIC	-	NA	NA
	Corrigan et al., 2019 [45]	6 right	Adequate	Pearson s = 0.992, *p* = 0.008, v = 0.994, *p* = 0.006, d = 0.997, *p* = 0.003	+	NA	NA

*NA* Znot applicable, *SW* Shear wave, *MVIC* Maximum voluntary isometric contraction

The overall quality ratings for each article assessing these measurement properties were predominantly adequate. Articles rated as doubtful are reported at this stage but will not be included in the following phase to combine results for best-quality evidence. These doubtful articles had non-optimal statistical analyses or a lack of proper reporting regarding the blinding of assessors. Refer to Appendix 4 for a detailed explanation of Tables 4 and 5.

### Best evidence synthesis

Given the large variety between methodologies and the identified inconsistencies within each type of elastography results were combined cautiously. Appendix 5 provides a detailed account of the methodologies applied in the included articles. Some of the major differences found included patient positioning with the ankle either relaxed or set at a specific dorsiflexed or plantarflexed angle; the investigated part of the Achilles tendon, including the middle part of the free tendon, the myotendinous junction, level of the medial malleolus and specific areas such as 5 cm from the enthesis. Other identified differences included the type of the pre-set used on the ultrasonographic machine, whether online or offline processing was carried out, the probe placement including longitudinal and transverse planes, and the placement of the region of interest on the tendon to be assessed. These findings will be explored further in the first part of the discussion section, where each elastography modality evidence is analysed.

Tables 6, 7 and 8 present the grading of evidence for reliability, validity and measurement error respectively for the different elastography methods and a summary of the rating of good measurement properties according to the statistical results of the articles which obtained an adequate or very good rating for the ROB. Evidence for some measurement properties is indetermined, either because there was no information available or available from one study as obtained for the reliability of shear wave modulus and 3D SWE. Findings of measurement error were mostly indeterminate scores, which do not suggest that the measurement instrument is of poor quality, but only highlight the need for more high-quality studies that can adequately assess the measurement properties. The best evidence synthesis for each elastography method will be reported separately in the next section.

**Table 6:** Reliability evidence grading

Reliability		Summary or pooled ICC result	Overall rating of good measurement properties	Quality of evidence
**Strain elastography**	Intra rater same day	Total sample size—176	Sufficient	**Moderate** (downgraded for inconsistency)
		**Longitudinal** > 0.7		
		Total sample size—58	Sufficient	**Moderate** (downgraded for imprecision sample size < 100)
		**Transverse** < 0.45		
	Intra rater different days	Total sample size—20	Insufficient	/
		**Longitudinal** > 0.1–0.87		
		**Transverse** > 0.1		
	Inter-rater	Total sample size—110	Sufficient	**Moderate** (downgraded for inconsistency)
		**Longitudinal** = 0.51–0.79		
		Assessed in **one article**	Insufficient	/
		Total sample size—50		
		Transverse = 0.41		
**Shear wave velocity**	Intra rater same day	Assessed in **one article**		/
		Total sample size—14		
		**Longitudinal** = 0.55–0.67	Insufficient	
		**Transverse** = 0.78–0.85	Insufficient	
	Intra rater different days	Total sample size—36	Sufficient	**Very Low** (downgraded 2 for imprecision sample size < 50 & inconsistency)
		**Longitudinal** = 0.54–0.71		
		Assessed in **one article**	Insufficient	/
		Total sample size—14		
		**transverse** = 0.62–0.71		
	Inter-rater	Total sample size—665	Sufficient	**Moderate** (downgraded for inconsistency)
		**Longitudinal** = 0.455–0.923		
		Total sample size—655	Sufficient	**Moderate** (downgraded for indirectness)
		**Transverse** = 0.7–0.87		
**Shear wave modulus**	Intra rater same day	Assessed in **one article**	Insufficient	/
		Total sample size—7		
		**Compression modulus** = 0.42–0.85		
	Intra rater different days	Assessed in **one article**	Insufficient	/
		Total sample size—20		
		**Shear modulus** = 0.82–0.88		
	Inter-rater	Assessed in **one article**	Insufficient	/
		Total sample size—20		
		**Shear modulus** = 0.77–0.93		
**cSWE**	Intra rater same day	Total sample size—78	Sufficient	**Low** (downgraded for imprecision sample size < 100 and indirectness)
		**shear modulus** = 0.7–0.88		
		**viscosity modulus** = 0.87–0.88		
	Intra rater different days	Assessed in one article	Insufficient	/
		Total sample size—20		
		**shear modulus** = 0.7		
		**viscosity modulus** = 0.87		
**3D SWE**	Intra rater different days	Assessed in **one article**	Insufficient	/
		Total sample size—10		
		0.59		
	Inter-rater	Assessed in **one article**	Insufficient	/
		Total sample size—10		
		0.44

**Table 7:** Validity evidence grading

Validity		Summary or pooled result	Overall rating of good measurement properties	Quality of evidence
**Strain elastography**	Convergent	Assessed in **one article**	Insufficient	/
		Correlated to VISA-A for symptomatic AT		
		Total sample size—240		
		Assessed in **one article**	Insufficient	/
		Correlated to B mode US		
		Total sample size—240		
		Correlated to isometric contractions	Sufficient	**Moderate** (downgraded for indirectness)
		Total sample size—114		
	Discriminative	Assessed in **one article**	Insufficient	/
		Age		
		Total sample size 100		
		Gender	Sufficient	**Moderate** (downgraded for indirectness)
		Total sample size—114		
**Shear wave velocity**	Convergent	Correlated to VISA-A for symptomatic AT	Sufficient	**High**
		Total sample size—207		
	Discriminative	age	Sufficient	**High**
		Total sample size—702		
		Assessed in **one article**	Insufficient	/
		Gender		
		Total sample size—652		
		Tendinopathy	Sufficient	**Moderate** (downgraded for inconsistency)
		Total sample size—342		
		Foot posture—increases with dorsiflexion	Sufficient	**High**
		Total sample size—370		
		Assessed in** one article**	Insufficient	/
		BMI		
		Total sample size—50		
**Shear wave modulus**	Criterion	Assessed in **one article**	Insufficient	/
		Correlated to MRI, US, doppler flow & UTC		
		Total sample size—75		
	Convergent	Assessed in **one article**	Insufficient	/
		correlated to dispersion analysis		
		Total sample size—10		
		Correlated to isometric contraction	Sufficient	**Low** (downgraded for imprecision sample size & indirectness)
		Total sample size—88		
**cSWE**	Convergent	correlation to MVIC	Sufficient	**Very Low** (downgraded for imprecision sample size < 100 & inconsistency & indirectness)
		Total sample size—58		
		Pearson CC = < 0.12–0.99		
		Assessed in **one article**	Insufficient	/
		Correlation to shear wave modulus		
		Total sample size—6		

*VISA-A* Victorian institute of sport assessment-achilles, *US* Ultrasound, *MRI* Magnetic resonance imaging, *UTC* Ultrasound tissue characterization, *MVIC* Maximum voluntary isometric contraction, *CC* Correlation coefficient

**Table 8:** Measurement Error evidence grading

Measurement error	Summary or pooled result	Overall rating	Quality of evidence
**Strain elastography**	No MIC reported	Indeterminate	/
	Total sample size—108		
**Shear wave velocity**	No MIC reported	Indeterminate	/
	Total sample size—24		
**Shear wave modulus**	SEM = 11.87–21.75 kPa	Indeterminate	/
	Total sample size—27		
**cSWV**	SEM s = 3.8–8.28 kPa v = 4.79–6.8Pas d = 46.72 kPa	Indeterminate	/
	Total sample size—78		
**3D SWV**	Assessed in **one article**	Insufficient	/
	Total sample size—108		

*MIC* Minimal important change, *SEM* Standard error of measure, *s* Shear modulus, *v* Viscosity modulus, *d* Dynamic modulus

### Strain elastography

Construct validity of strain ratio received positive ratings for correlation to both VISA-A and ultrasonographic imaging when assessing tendinopathic participants. Since only one study investigated this correlation, grading of evidence was not conducted. The strain ratio correlated to isometric contraction obtained a moderate level of evidence, with the ability to significantly detect a decrease in this ratio (i.e. tendon becomes harder since the reference material remains the same through the measurement) with the increase in loading. The intra-rater reliability of the strain ratio was rated as having a moderate level of evidence, for both longitudinal and transverse probe placements. However, the former obtained positive results, with only one article showing low ICC, while the latter obtained only negative ratings within a combined sample size of only 58 participants. Inter-rater reliability was downgraded to moderate for the longitudinal probe placement due to inconsistencies in results.

### Shear wave velocity

Convergent validity of SWV was correlated to the patient-reported outcome measure, the VISA-A, when tendinopathy was being assessed and was graded as high evidence. For discriminative validity, SWV was able to measure significant differences in foot positions and age, both receiving a high level of evidence, while moderate evidence was found when differentiating between pathological and healthy tendons. The quality of evidence for inter-rater reliability was rated as moderate due to inconsistency in results when using a longitudinal probe position and indirectness when using a transverse position. Intra-rater reliability within the same day was not graded, as only one article was found. However, when intra-rater reliability was assessed in different sessions, mixed results were present, with some articles having an ICC of 0.71 while others reported a lower value of 0.54. This conclusion was based on a total sample size of only 36 participants.

### Shear wave modulus

No grading of evidence was possible for the criterion validity of SWE as only one article assessed the correlation to MRI, B-mode ultrasound, power Doppler, and ultrasonographic tissue characterisation (UTC). A good correlation between instruments was found for diagnostic accuracy. When convergent validity was assessed, no correlation was found between shear wave modulus and isometric contraction in the two articles. Thus, the grading of evidence was downgraded to low due to the small healthy sample size on which the results are based. Insufficient data is present for all types of reliability assessed when using shear wave modulus and therefore no grading of evidence was conducted. Responsiveness was only investigated in one article and SW modulus showed a significant change when a 6-month follow-up was compared to baseline data. However, only poor monitoring accuracies were found for midportion Achilles tendinopathy so evidence could not be graded.

### Continuous shear wave elastography

Convergent validity of cSWE to maximum voluntary isometric contraction (MVIC) obtained inconsistent results within a small sample size of healthy people; thus grading of evidence was downgraded to very low. Only one article studied the correlation to shear wave modulus, so evidence was not graded. A low level of evidence is present for intra-rater same-day cSWE reliability due to the indirectness of the study population while inter-rater was not investigated.

#### Three-dimensional Shear wave elastography

Evidence was not graded for 3D SWE as only one article was found investigating this method. Moreover, no validity testing in vivo was conducted.

## Discussion

This systematic review identified four different modalities of elastography: strain elastography or also known as compression elastography, shear wave elastography, continuous shear wave elastography, and 3D elastography. Each elastography method will first be discussed in light of the inconsistencies (Appendix 5) and quality of evidence collated on the identified measurement properties as presented in Table 6 for reliability, Table 7 for validity, and Table 8 for measurement error). General considerations that should be taken into account when evaluating the evidence of each measurement property of different elastography modalities will also be discussed.

### Strain elastography

Strain elastography is considered a semi-quantitative measure, represented as a ratio of tissue stiffness in comparison to its surrounding or external reference material. This strain index can be used as a comparative index and should not be considered an absolute strain measurement. The use of different reference materials led to both high and low ICC values, leading to mixed positive and negative results, especially when Kager’s fat pad was the chosen comparator for patients with Achilles tendinopathy. Material properties of the fat pad can change due to the pathology itself [49], thus giving rise to a false ratio. For this reason, it is recommended that when opting for strain elastography as a measurement instrument, an external reference material with known elastic properties is used. This will allow comparisons of strain ratios under different conditions and among subjects.

In this review, the validity and reliability of strain elastography obtained moderate ratings, supporting its use to measure the Achilles tendon material properties. It was found to be a highly operator-dependent procedure with better reliability in more experienced professionals. Repeated manual compression using the ultrasound transducer causes axial strain in the tissue of interest. These compressions produce a displacement within the tissue, which is less pronounced in harder than softer materials.

### Shear wave imaging – shear wave velocity and modulus

Shear wave imaging has the advantage of providing quantitative measures within a relatively small region of the tendon, thus tendon pathology which is known to affect discrete areas, can be accurately identified and measured. It is believed that using shear wave modalities is more reliable than strain elastography as the compressions are automatically induced by using a radiation force of ultrasound beams [50]. However, the grading of evidence from this review suggests that reliability properties are still low and insufficient to arrive at such conclusions.

Direct comparison of shear wave imaging results was not possible as some articles based their analysis on estimates of Young’s modulus (E) rather than reporting the underlying shear wave velocity. These two variables are directly related but not the same [51, 52]. Converting SWV to E relies on the equation E = 3 pv^2^ where v is SWV and p is tissue density [47, 50]. This equation assumes tissue isotropy based on 1000 kg.m^3^ used as a constant tissue density; however, this may not always be true as tendons are found to be heterogeneous [53], anisotropic [54] and viscoelastic [55], with variation in their structural composition and fluid consistency especially when pathology is present [56]. Thus, it is recommended that shear wave velocity should be reported rather than the shear modulus.

Although criterion validity against the gold standard method of dynamometry and ultrasonography was not investigated, several studies found shear wave imaging to be a valid tool to differentiate between healthy and diseased tendons, as tendinopathic tendons are significantly less stiff than healthy ones [29]. Results also correlate strongly to the patient-reported outcome measure; the VISA-A questionnaire and clinical symptoms make shear wave imaging a valid tool to identify tendon damage with high evidence.

One of the major drawbacks of shear wave imaging is that it can result in saturation of the elastogram in ankle positions around 0° of flexion since the Achilles tendon bears high tensile loads in this position or when dorsiflexed. Given this limitation, authors [38] of previous research suggested that the evaluation of AT should be limited to a relaxed or plantar flexed position and not stretched or loaded tendon with additional weight as this will increase the shear wave velocity [39, 57] leading to saturation and possibly false results.

Inconsistencies were also present when calculating the shear wave velocity and modulus. The midportion of the tendon was investigated at different lengths from 1 cm from the insertion up to the myotendinous junction of the soleus muscle and the gastrocnemius myotendinous junction [39]. Furthermore, no homogeneity exists on how to identify the region of interest (ROI) to measure the shear wave velocity. Some authors based their findings on the whole thickness of the tendon, while others applied multiple circular or box ROIs of 1 to 3 mm and an average was taken within the same frame or different frames of a recorded clip for offline analysis. In a study assessing the size of ROI, transducer pressure, and time of acquisition [58], the authors found significant differences in the maximum value of the elastic modulus of the rectus femoris and patellar tendon when different ROI sizes were used. Unfortunately, no consensus exists on which protocol works best to improve reliability and with the limited available literature present and the high heterogeneity that exists, recommendations cannot be made. Further research on these technical aspects can identify whether the same results are achieved when using different methods especially when diseased tendons are measured. Recent reporting guidelines on the use of shear wave elastography [59] suggest that the region of interest information should be reported in detail, including the position, number, size of the ROIs and whether ROIs were standardised and kept constant across all participants.

### Continuous shear wave elastography

cSWE overcomes the issue of saturation by using an external actuator to generate shear waves across a specified range of frequencies. However, this recent innovation is still in its infancy stage and insufficient literature is available to grade data. One limitation is that it requires an extra pair of hands due to the added actuator that is placed near the probe.

#### Three-dimensional Shear wave elastography

3D SWE has been recently introduced to better acquire a three-dimensional acquisition volume of the tendon’s stiffness. However, its validity remains questionable because of anisotropy when tendon fibres are not perfectly aligned. Advancements in ultrasound transducers allowed for reduced time for the elastogram to stabilise thus reducing the effects of movement artefact when obtaining results. However, any conclusions are premature, inconclusive, and further research is needed to explore the measurement properties of this method.

### Considerations on reliability, validity, measurement error, and responsiveness

#### Reliability and validity

Most of the above gradings of evidence were based on studies investigating healthy subjects. Although this is critical for establishing reliability and validity, uncertainty remains in the presence of tendon injury. Reliability depends on the homogeneity of the study population being assessed, affecting the generalisability of results. Homogeneity reduces the variance between participants, and the ICC values will be a conservative estimate of the reliability to be expected in a cohort more representative of the general population.

The importance of having reliable instruments available becomes increasingly essential since the translation of data into clinical practice is safer and more accurately reflects the functional condition of the evaluated person. It was evident that a standardised protocol optimises reliability because repeated measurements are similar and the error of measurement arising from variation in measurement protocols is kept to a minimum. This review reported the methodological inconsistencies found that hinder further analysis of results. Standardised ultrasonographic technical settings and positioning of the patient with monitored muscle activity are imperative for better interpretable results.

An important consideration that was missing in some of the assessed reliability articles was the preconditioning of the tendon. Since tendons have elastic [60] and viscoelastic properties, with other time-dependent mechanical properties affected by loading history [48] and hydration state [61], pre-conditioning protocols should be used to ensure that the tendon behaves in a repeatable way.

### Measurement error

The overall evidence for measurement error could not be determined because assessing measurement error takes into consideration intra-individual variability between repeated measurements and is often expressed as the coefficient of variation, the smallest detectable change, and the limits of agreement. An instrument with a large error of measurement may fail to detect the true meaningful change in an individual patient.

Understanding if a truly meaningful change has occurred, is of added value. This meaningful change has clinical importance in identifying an improvement in physical functioning that is large enough for the person to perceive a difference [62, 63]. If no minimal important change (MIC) is reported in studies, there is no value upon which to make a comparison, and the measures by which a meaningful change is judged may not reflect the true state. There is still ongoing debate about how the MIC should be assessed [25] and so graded evidence of the measurement error was indeterminate for all elastography modalities. This value also varies according to context, as MIC derived from study participants who are healthy may have limited value for studies that investigate participants with tendinopathy.

### Responsiveness

Evidence on responsiveness cannot be determined as very few articles were found to consider this measurement property. Only one article using axial strain elastography and another using SWE having a longitudinal design were found to determine the changes that are occurring over a period of time. This shows that no established measurement instrument is yet identified as a treatment monitoring instrument, which aggravates the detection of early treatment effects or possible complications.

## Conclusions

This systematic review explored and highlighted the paucity of evidence for the measurement properties of different elastography methods. Our data synthesis focused on the qualitative approach as considerable heterogeneity between studies was present, thus not allowing for a meta-analysis of results. The qualitative approach adopted permitted the best possible synthesis of evidence that accounted for between-study similarities, the quality of each study, and the consistency of measurement properties reported across different studies. There are a limited number of studies exploring quantitative elastography on Achilles tendinopathy as most evidence found in this review was based on a healthy population. Only articles published in the English language were eligible for inclusion. It is, therefore, possible that other potential studies might have been excluded.

Based on the identified evidence on the measurement properties of elastography, none of the different elastography methods showed superiority over the others with gradings ranging from very low to moderate. However, strain elastography and shear wave elastography reported as shear wave velocity have the best potential to be used in the identification of tendinopathy. Further high-quality studies with robust longitudinal designs to investigate responsiveness are needed to aid the monitoring of tendon recovery and detect any differences over time that may be attributed to true physiological changes.

## Supplementary Information

Additional file 1: Appendix 1. Search strategy keywords.

Additional file 2: Appendix 2. Search Strategies.

Additional file 3. Appendix 3

Additional file 4: Appendix 4. Strain elastography.

Additional file 5: Appendix 5. Articles methodology inconsistencies.

## Data Availability

Authors are happy to share the data for this work on request. No reprints are available.

## Abbreviations

AT: Achilles tendinopathy
US: Ultrasonography
PRISMA: Preferred reporting items for systematic reviews and meta-analyses
COSMIN: COnsensus-based standards for the selection of health measurement i**n**struments
ROB: Risk of bias
GRADE: Grading of recommendations assessment, development, and evaluation
SSI: Supersonic shear wave imaging
SWV: Shear wave velocity
cSWE: Continuous shear wave elastography
3D SWE: Three-dimensional shear wave elastography
VISA-A: Victorian institute of sport assessment-achilles
ICC: Intraclass correlation coefficient
UTC: Ultrasonographic tissue characterisation
MVIC: Maximum voluntary isometric contraction
MIC: Minimal important change
ROI: Region of interest
