# Supplementary material for: Elastography in the assessment of the Achilles tendon: a systematic review of measurement properties
Source: J Foot Ankle Res. 2023 Apr 27;16:23. doi: 10.1186/s13047-023-00623-1 (PMC10134611; doi:10.1186/s13047-023-00623-1)
Supplement: Supplementary file 1 — Additional file 1: Appendix 1. Search strategy keywords. [file 13047_2023_623_MOESM1_ESM.docx]

| **Construct search** | **Target population search** | **Instrument search** |
| --- | --- | --- |
| Shear wave velocity | Achilles tendon | Elastography |
| Shear wave modulus | Triceps surae | Strain elastography |
| Strain ratio | Calcaneal tendon | 3D elastography |
| Mechanical properties | Gastrocnemius-soleus complex | continuous shear wave elastography |
| Material properties | Tendinopathy | Sonoelastography |
| Youngs modulus | Tendinosis |  |
|  | Tendon injury |  |

**Appendix 1 – Search strategy keywords**
