# Supplementary material for: Elastography in the assessment of the Achilles tendon: a systematic review of measurement properties
Source: J Foot Ankle Res. 2023 Apr 27;16:23. doi: 10.1186/s13047-023-00623-1 (PMC10134611; doi:10.1186/s13047-023-00623-1)
Supplement: Supplementary file 2 — Additional file 2: Appendix 2. Search Strategies. [file 13047_2023_623_MOESM2_ESM.docx]

**Appendix 2 -  Search Strategies**

#1  **construct search** - S1- shear wave velocity OR shear wave modulus OR strain ratio OR mechanical properties OR Youngs modulus

#2   **target population search** *- S2* - Achilles tendon OR calcaneal tendon OR triceps surae OR gastrocnemius-soleus complex OR tendinopathy OR tendinosis OR tendon injury

#3   **instrument search** - S3 – elastography OR strain elastography OR 3D elastography OR Shear wave elastography OR continuous shear wave elastography OR sosnoelstography

**PubMed**

#4  #1 AND #2 AND #3 AND **measurement properties** filter - (This filter has a sensitivity of 97.4% and a precision of 4.4% (Terwee et al., 2009)) S4 - (instrumentation[sh] OR methods[sh] OR “Validation Studies”[pt] OR “Comparative Study”[pt] OR “psychometrics”[MeSH] OR psychometr*[tiab] OR clinimetr*[tw] OR clinometr*[tw] OR “outcome assessment (health care)”[MeSH] OR “outcome assessment”[tiab] OR “outcome measure*”[tw] OR “observer variation”[MeSH] OR “observer variation”[tiab] OR “Health Status Indicators”[Mesh] OR “reproducibility of results”[MeSH] OR reproducib*[tiab] OR “discriminant analysis”[MeSH] OR reliab*[tiab] OR unreliab*[tiab] OR valid*[tiab] OR “coefficient of variation”[tiab] OR coefficient[tiab] OR homogeneity[tiab] OR homogeneous[tiab] OR “internal consistency”[tiab] OR (cronbach*[tiab] AND (alpha[tiab] OR alphas[tiab])) OR (item[tiab] AND (correlation*[tiab] OR selection*[tiab] OR reduction*[tiab])) OR agreement[tw] OR precision[tw] OR imprecision[tw] OR “precise values”[tw] OR test-retest[tiab] OR (test[tiab] AND retest[tiab]) OR (reliab*[tiab] AND (test[tiab] OR retest[tiab])) OR stability[tiab] OR interrater[tiab] OR inter-rater[tiab] OR intrarater[tiab] OR intra-rater[tiab] OR intertester[tiab] OR inter-tester[tiab] OR intratester[tiab] OR intra-tester[tiab] OR interobserver[tiab] OR inter-observer[tiab] OR intraobserver[tiab] OR intra-observer[tiab] OR intertechnician[tiab] OR inter-technician[tiab] OR intratechnician[tiab] OR intra-technician[tiab] OR interexaminer[tiab] OR inter-examiner[tiab] OR intraexaminer[tiab] OR intra-examiner[tiab] OR interassay[tiab] OR inter-assay[tiab] OR intraassay[tiab] OR intra-assay[tiab] OR interindividual[tiab] OR inter-individual[tiab] OR intraindividual[tiab] OR intra-individual[tiab] OR interparticipant[tiab] OR inter-participant[tiab] OR intraparticipant[tiab] OR intra-participant[tiab] OR kappa[tiab] OR kappa’s[tiab] OR kappas[tiab] OR repeatab*[tw] OR ((replicab*[tw] OR repeated[tw]) AND (measure[tw] OR measures[tw] OR findings[tw] OR result[tw] OR results[tw] OR test[tw] OR tests[tw])) OR generaliza*[tiab] OR generalisa*[tiab] OR concordance[tiab] OR (intraclass[tiab] AND correlation*[tiab]) OR discriminative[tiab] OR “known group”[tiab] OR “factor analysis”[tiab] OR “factor analyses”[tiab] OR “factor structure”[tiab] OR “factor structures”[tiab] OR dimension*[tiab] OR subscale*[tiab] OR (multitrait[tiab] AND scaling[tiab] AND (analysis[tiab] OR analyses[tiab])) OR “item discriminant”[tiab] OR “interscale correlation*”[tiab] OR error[tiab] OR errors[tiab] OR “individual variability”[tiab] OR “interval variability”[tiab] OR “rate variability”[tiab] OR (variability[tiab] AND (analysis[tiab] OR values[tiab])) OR (uncertainty[tiab] AND (measurement[tiab] OR measuring[tiab])) OR “standard error of measurement”[tiab] OR sensitiv*[tiab] OR responsive*[tiab] OR (limit[tiab] AND detection[tiab]) OR “minimal detectable concentration”[tiab] OR interpretab*[tiab] OR ((minimal[tiab] OR minimally[tiab] OR clinical[tiab] OR clinically[tiab]) AND (important[tiab] OR significant[tiab] OR detectable[tiab]) AND (change[tiab] OR difference[tiab])) OR (small*[tiab] AND (real[tiab] OR detectable[tiab]) AND (change[tiab] OR difference[tiab])) OR “meaningful change”[tiab] OR “ceiling effect”[tiab] OR “floor effect”[tiab] OR “Item response model”[tiab] OR IRT[tiab] OR Rasch[tiab] OR “Differential item functioning”[tiab] OR DIF[tiab] OR “computer adaptive testing”[tiab] OR “item bank”[tiab] OR “cross-cultural equivalence”[tiab]).

#5   #4 NOT **exclusion** filter - S5 - (“addresses”[Publication Type] OR “biography”[Publication Type] OR “case reports”[Publication Type] OR “comment”[Publication Type] OR “directory”[Publication Type] OR “editorial”[Publication Type] OR “festschrift”[Publication Type] OR “interview”[Publication Type] OR “lectures”[Publication Type] OR “legal cases”[Publication Type] OR “legislation”[Publication Type] OR “letter”[Publication Type] OR “news”[Publication Type] OR “newspaper article”[Publication Type] OR “patient education handout”[Publication Type] OR “popular works”[Publication Type] OR “congresses”[Publication Type] OR “consensus development conference”[Publication Type] OR “consensus development conference, nih”[Publication Type] OR “practice guideline”[Publication Type]) NOT (“animals”[MeSH Terms]

**CLINAHL**

#4  #1 AND #2 AND #3 AND **filter for measurement properties**

(MH “Psychometrics”) or ( TI psychometr* or AB psychometr* ) or ( TI clinimetr* or AB clinimetr* ) or ( TI clinometr* OR AB clinometr* ) or (MH “Outcome Assessment”) or ( TI outcome assessment or AB outcome assessment ) or ( TI outcome measure* or AB outcome measure* ) or (MH “Health Status Indicators”) or (MH “Reproducibility of Results”) or (MH “Discriminant Analysis”) or ( ( TI reproducib* or AB reproducib* ) or ( TI reliab* or AB reliab* ) or ( TI unreliab* or AB unreliab* ) ) or ( ( TI valid* or AB valid* ) or ( TI coefficient or AB coefficient ) or ( TI homogeneity or AB homogeneity ) ) or ( TI homogeneous or AB homogeneous ) or ( TI “coefficient of variation” or AB “coefficient of variation” ) or ( TI “internal consistency” or AB “internal consistency” ) or (MH “Internal Consistency+”) or (MH “Reliability+”) or (MH “Measurement Error+”) or (MH “Content Validity+”) or “hypothesis testing” or “structural validity” or “cross-cultural validity” or (MH “Criterion-Related Validity+”) or “responsiveness” or “interpretability” or ( TI reliab* or AB reliab* ) and ( (TI test or AB test) OR (TI retest or AB retest) ) or ( TI stability or AB stability ) or ( TI interrater or AB interrater ) or ( TI inter-rater or AB inter-rater ) or ( TI intrarater or AB intrarater ) or ( TI intra-rater or AB intrarater ) or ( TI intertester or AB intertester) or (TI inter-tester or AB inter-tester) or ( TI intratester or AB intratester) or ( TI intra-tester or AB intra-tester) or ( TI interobserver or AB interobserver) or (TI inter-observer or AB inter-observer ) or ( TI intraobserver or AB intraobserver) or ( TI intra-observer or AB intra-observer) or ( TI intertechnician or AB intertechnician) or (TI inter-technician or AB inter-technician) or ( TI intratechnician or AB intratechnician ) or ( TI intra-technician or AB intra-technician ) or ( TI interexaminer or AB interexaminer ) or (TI inter-examiner or AB inter-examiner) or (TI intraexaminer or AB intraexaminer ) OR (TI intra-examiner or AB intra-examiner ) or (TI intra-examiner or AB intraexaminer ) or (TI interassay or AB interassay ) or ( TI inter-assay or AB inter-assay ) or ( TI intraassay or AB intraassay) or ( TI intra-assay or AB intra-assay ) or (TI interindividual or AB interindividual) or (TI inter-individual or AB inter-individual) OR (TI intraindividual or AB intraindividual) or (TI intra-individual or AB intra-individual) or (TI interparticipant or AB interparticipant) or (TI inter-participant or AB inter-participant ) or (TI intraparticipant or AB intraparticipant) or (TI intra-participant or AB intra-participant ) or (TI kappa or AB kappa) or (TI kappa’s or AB kappa’s ) or (TI kappas or AB kappas) or (TI repeatab* or AB repeatab*) or ( TI responsive* or AB responsive* ) or ( TI interpretab* or AB interpretab* )

**OVID MEDLINE**

#4  #1 AND #2 AND #3 AND

1. (instrumentation or methods).fs.

2. (Validation Studies or Comparative Study).pt.

3. exp Psychometrics/

4. psychometr*.ti,ab.

5. (clinimetr* or clinometr*).tw.

6. exp “Outcome Assessment (Health Care)”/

7. outcome assessment.ti,ab.

8. outcome measure*.tw.

9. exp Observer Variation/

10. observer variation.ti,ab.

11. exp Health Status Indicators/

12. exp “Reproducibility of Results”/

13. reproducib*.ti,ab.

14. exp Discriminant Analysis/

15. (reliab* or unreliab* or valid* or coefficient or homogeneity or homogeneous or “internal consistency”).ti,ab.

16. (cronbach* and (alpha or alphas)).ti,ab.

17. (item and (correlation* or selection* or reduction*)).ti,ab.

18. (agreement or precision or imprecision or “precise values” or test-retest).ti,ab.

19. (test and retest).ti,ab.

20. (reliab* and (test or retest)).ti,ab.

21. (stability or interrater or inter-rater or intrarater or intra-rater or intertester or inter-tester or intratester or intra-tester or interobserver or inter-observer or intraobserver or intraobserver or intertechnician or inter-technician or intratechnician or intra-technician or interexaminer or inter-examiner or intraexaminer or intra-examiner or interassay or interassay or intraassay or intra-assay or interindividual or inter-individual or intraindividual or intra-individual or interparticipant or inter-participant or intraparticipant or intra-participant or kappa or kappa’s or kappas or repeatab*).ti,ab.

22. ((replicab* or repeated) and (measure or measures or findings or result or results or test or tests)).ti,ab.

23. (generaliza* or generalisa* or concordance).ti,ab.

24. (intraclass and correlation*).ti,ab.

25. (discriminative or “known group” or factor analysis or factor analyses or dimension* or subscale*).ti,ab.

26. (multitrait and scaling and (analysis or analyses)).ti,ab.

27. (item discriminant or interscale correlation* or error or errors or “individual variability”).ti,ab.

28. (variability and (analysis or values)).ti,ab.

29. (uncertainty and (measurement or measuring)).ti,ab.

30. (“standard error of measurement” or sensitiv* or responsive*).ti,ab.

31. ((minimal or minimally or clinical or clinically) and (important or significant or detectable) and (change or difference)).ti,ab.

32. (small* and (real or detectable) and (change or difference)).ti,ab.

33. 1 or 2 or 3 or 4 or 5 or 6 or 7 or 8 or 9 or 10 or 11 or 12 or 13 or 14 or 15 or 16 or 17 or 18 or 19 or 20 or 21 or 22 or 23 or 24 or 25 or 26 or 27 or 28 or 29 or 30 or 31 or 32
