# Supplementary material for: Elastography in the assessment of the Achilles tendon: a systematic review of measurement properties
Source: J Foot Ankle Res. 2023 Apr 27;16:23. doi: 10.1186/s13047-023-00623-1 (PMC10134611; doi:10.1186/s13047-023-00623-1)
Supplement: Supplementary file 3 — Additional file 3. Appendix 3 [file 13047_2023_623_MOESM3_ESM.docx]

**Appendix 3**

| Element | Instruction | Study |
| --- | --- | --- |
| 1. Name of the  Instrument | Alternatively:  type of  instrument and  Parameter | Supersonic shear wave - Shear wave elastography |
| 2. Version or  way of  operationalization | All relevant  components that  are known or  expected to  influence the  score, and which  are standardized  or restricted  (facet of  stratification | **Equipment** AIXPLORER (v.9, Supersonic Image, Aix-en-Provence, France) was used for the shear modulus maps acquisition with two linear transducers operating in the frequencies of 4-15 MHz and 2-10 MHz  **Preparatory actions:** The volunteers were positioned lying prone and relaxed, for the MG and AT images acquisition. Images were acquired bilaterally and twice for each structure by a single ultrasound-experienced operator. |
|  |  | Preparation: They were instructed not to perform heavy training between sessions. The exclusion criteria were any report of pain, myotendinous lesion or previous surgery in the structures of interest.  First, the transducer was longitudinally positioned  over the AT, observing the proximal AT insertion at the right side of the rectangle map (region of interest). Subsequently, the transducer was  positioned over the MG muscle, longitudinally to the limb, in the region of 30% of the proximal  leg length (distance between the fibular head and lateral malleolus). The gel (Ultrex-gel, Farmative Industry and Trade Ltda., Brazil) was used for acoustic coupling on the surface of the skin.  The isometric plantarflexion torque was tested with  the isokinetic dynamometer (Biodex System 4 Pro,  New York, USA). Subjects were seated on the dynamometer chair, with 90° of hip flexion, full knee extension (avoiding hyperextension) and ankle at 90° (neutral position) fixed on the foot platform. After protocol familiarization, two 5-second maximal voluntary contractions (MVC) were performed at a 40-seconds interval. The maximum torque reached in each session was considered for analysis.  Unprocessed data  Collection: For the elastographic mode it was selected the musculoskeletal (MSK) or tendon presets, with scales ranging from 0-300 kPa and 0-800 kPa, respectively.  A square area of approximately 1.00 cm side for the muscle and a rectangle of 1 x 4 cm for the tendon were selected for the elastographic colour mapping. The shear modulus maps were captured after 10 seconds to guarantee map stabilization. |
| 3. Construct | Description of  what is being  Measured | **Youngs modulus** -  For an isotropic and purely elastic medium, the shear wave propagation velocity (Cs) and the medium density (p = 1000 kg • m-3 for biological tissues) are related to E values according to the equation (1):E = 3 ·ρ ·(cs2) |
| 4. Measurement  property |  | Reliability – intra-rater same day & intra-rater different days |
| 5. Components  that will be  repeated | Either the whole  measurement  (i.e. all components) or the assignment  of the score (i.e.  last component) | The subjects were tested twice, with a one-week interval.  Whole measurements - A total of 16 images was obtained for each subject (two for each  structure, bilaterally, two session days visits)  The entire protocol was repeated in a second session, with a maximum interval of one week. For isokinetic torque measurements |
| 6. Source(s) of  variation varied | Components  which is varied  across the  measurements  (i.e. focus of  analysis; facet of  generalizability | by single ultrasound experienced operator  Minimal manual pressure was applied on the individual’s skin |
| 7. Patient  population | (i.e. facet of  differentiation) | 24 healthy male volunteers (28 ± 2 years,  88.4 ± 11.4 kg, and 1.80 ± 0.08 m) |
