# Supplementary material for: Elastography in the assessment of the Achilles tendon: a systematic review of measurement properties
Source: J Foot Ankle Res. 2023 Apr 27;16:23. doi: 10.1186/s13047-023-00623-1 (PMC10134611; doi:10.1186/s13047-023-00623-1)
Supplement: Supplementary file 4 — Additional file 4: Appendix 4. Strain elastography. [file 13047_2023_623_MOESM4_ESM.docx]

Appendix 4 -

## Strain elastography

Strain elastography was validated in four articles (28,35-37) which all scored a very good rating when compared to ultrasonography, other elastography methods and VISA-A. According to the statistical results, ratings of good measurement properties received both positive (28,36,37) and negative (35) ratings.

Reliability of strain elastography was analysed in five articles (28,33-35,38) all receiving an adequate to very good rating except for the inter-rater reliability in (35) which was rated as doubtful as no evidence provided that no systematic error has occurred. When the statistical results of each article were compared to the rating of good measurement properties, as mentioned in the methodology section, intra rater within the same day had positive results but only one study assessed intra rater on different days (33). Inter-rater reliability received both positive (34) and negative results (28) for the longitudinal probe placing while inter-rater reliability for transverse probe placing was only assessed in one article (34).

Measurement error of strain elastography (33-35), received an adequate ROB but ratings were indeterminate as no MIC was reported in the literature. Responsiveness (35) received both a very good COSMIN rating and a positive rating for good measurement properties.

Shear wave imaging

Shear wave Imaging was investigated in 13 articles. Eight reported results as shear wave velocity (29-31,39-42,48) while five (32,37,43-45) reported Young’s modulus. Validation was assessed in all articles except for Payne et al., (2018) and Gotschi et al., (2021) and all articles had a robust methodology, thus received an adequate to very good COSMIN rating. Criterion validity was only conducted in one article (32) and it was rated as having a positive statistical result. Other articles’ validity by looking at correlations to other instruments (convergent validity) or the ability to identify different groups (discriminative validity). Convergent validity for both shear wave velocity and modulus as compared to VISA-A and isometric contraction was rated as positive. Discriminative validity for shear wave velocity obtained positive results for age, and foot posture (velocity increases on increased dorsiflexion) and both positive and negative results for the ability to identify tendinopathy.

When reliability was considered, doubtful COSMIN ROB was rated to two articles (39-40) assessing shear wave velocity while another article assessing modulus (43). These were excluded for further analysis. The other articles received an adequate to very good rating implying a low level of bias. Statistical results obtained both positive and negative ratings for inter-rater reliability, positive results for intra-rater different days and negative for intra-rater same day.

Measurement error was only assessed in five articles (41,43-45,48). These articles had a good methodology receiving an adequate COSMIN ROB except for one (43) which was graded as doubtful due to lack of blinding of observers which undoubtedly increases the risk for bias. Only one article (45) received a positive statistical rating score as the others all had indeterminate findings.

## cSWE

The two articles (46,47) that investigated cSWE, had adequate validity, reliability and measurement error on the COSMIN rating. When the statistical results were compared to the criteria for good measurement properties, validity received both positive and negative ratings, intra-rater reliability received positive ratings, while measurement error had both indeterminate (47) and positive ratings (46).

## 3DSWE

One article (48) assessed the reliability and measurement error of 3DSWE. Both measurement properties were adequate on the COSMIN risk of bias. Reliability was then graded as negative while measurement error was indeterminate.
